# Diagnostic accuracy evaluation of the conventional and molecular tests for Spinal Tuberculosis in a cohort, head-to-head study

**DOI:** 10.1038/s41426-018-0114-1

**Published:** 2018-06-20

**Authors:** Guirong Wang, Weijie Dong, Tinglong Lan, Jun Fan, Kai Tang, Yuan Li, Guangxuan Yan, Guanglu Jiang, Yifeng Ma, Yuanyuan Shang, Shibing Qin, Hairong Huang

**Affiliations:** 1 National Clinical Laboratory on Tuberculosis, Beijing Key Laboratory on Drug-resistant Tuberculosis Research, Beijing Chest Hospital, Capital Medical University, Beijing Tuberculosis and Thoracic Tumor Institute, Beijing, China; 2 Department of Orthopedics, Beijing Bone and Joint Tuberculosis Diagnosis and Treatment Center, Beijing Chest Hospital, Capital Medical University, Beijing Tuberculosis and Thoracic Tumor Institute, Beijing, China

## Abstract

Early diagnosis of spinal tuberculosis (TB) is hampered by the flaws of conventional tests. The aim of this study was to assess the value of new and existing molecular tests in a prospective, head-to-head cohort study. Specimens were consecutively collected from spinal TB suspects in four hospitals in Beijing, China. Smear, culture, histopathology, Xpert MTB/RIF (Xpert), and MeltPro TB assays were performed in parallel using the same specimen from each patient. Drug-susceptibility testing (DST) was conducted on the isolates recovered. In total, 438 suspects were recruited; 319 of them were diagnosed with spinal TB according to the composite reference standard (CRS), which was composed of clinical, laboratory, histopathological, and radiological examinations and 18 months of follow-up. Based on conventional testing, 74.29% of patients were classified as confirmed cases, which increased to 90.6% when Xpert outcomes were integrated. Further, 76.60% of probable and 45.71% of possible cases were re-classified as confirmed cases with Xpert. Xpert (85.27%) produced higher sensitivity than histopathology (73.04%), MeltPro TB (57.68%), culture (51.72%) and smear (24.45%) (all *P* <0.001). Xpert was 100% concordant with phenotypic DST regarding rifampicin resistance detection. The sensitivity and specificity of MeltPro TB for rifampicin resistance detection were 100% and 97.96%, respectively, and 95.00% and 93.88% for isoniazid resistance detection. New molecular tests demonstrated excellent efficiency for spinal TB diagnosis in this cohort study, so their application as initial diagnostic tools would greatly increase the proportion of confirmed cases and dramatically reduce the delay of appropriate treatment. An updated laboratory testing algorithm of the disease is desirable.

## Introduction

Tuberculosis (TB) is the ninth leading cause of death worldwide and the leading cause from a single infectious agent, with an estimated 10.4 million new TB cases in 2016^1^. It mostly affects lungs (pulmonary TB) but can affect other sites as well (extrapulmonary tuberculosis, EPTB). EPTB accounted for 15% of the 6.3 million incident cases that were documented by the WHO in 2016^1^. Spinal TB, one type of EPTB, is one of the most common and serious forms of TB lesions and accounts for 1-3% of all TB cases^2^. The incidence of spinal TB has been increasing^3^, probably as a consequence of aging populations, easy access to better diagnostics and an increased number of immunocompromised hosts^4^. Sequelae happen in 47.5% of spinal TB patients, and the mortality rate is 8.6%^5^.

Prognosis of spinal TB is largely influenced by early diagnosis, which leads to initiation of proper treatment^6^. Definitive diagnosis depends on the detection of *Mycobacterium tuberculosis* (Mtb) in the specimen, but the bacteriological evidence can only be acquired from 10 to 30% of the cases using conventional methods, e.g., smear and culture^7^. This limitation makes histopathological diagnosis critically important for diagnosing spinal TB. Although recognition of granulomatous inflammation by hematoxylin-eosin staining and identification of mycobacteria with Ziehl-Neelsen (ZN) staining provide etiological clues, the non-specific characteristic of granuloma and the low sensitivity of ZN staining remain the major limitations^8,9^. The WHO recommended Xpert MTB/RIF (Xpert) for EPTB diagnosis in 2013. Since then, several studies have evaluated its diagnostic accuracy for bone and joint TB^10–18^. According to these results, its acquired sensitivity is 69–100%, whereas the specificity is 16.6–100%. The MeltPro TB assay (Zeesan Biotecheh, Xiamen, China) is a melting curve analysis-based kit for detection of resistance to the main first-line and second-line anti-TB drugs^19^. Currently, it is widely used on respiratory samples in China. However, this method has not yet been evaluated vigorously with EPTB samples. Nucleic acid amplification tests (NAATs) are more sensitive and much faster than traditional tests, but their performances have varied between studies^20,21^. This inconsistency is mainly caused by small sample sizes and different study populations and designs. Moreover, most studies evaluated various methods using different samples of the enrolled patients, which could cause bias as well.

Despite the availability of multiple diagnostic methods, an appropriate algorithm for using such methods to diagnose spinal TB remains unavailable. For this reason, we conducted a multi-center, head-to-head cohort study for traditional, as well as new molecular tests to diagnose spinal TB.

## Results

### Patient characteristics

**Fig. 1:**
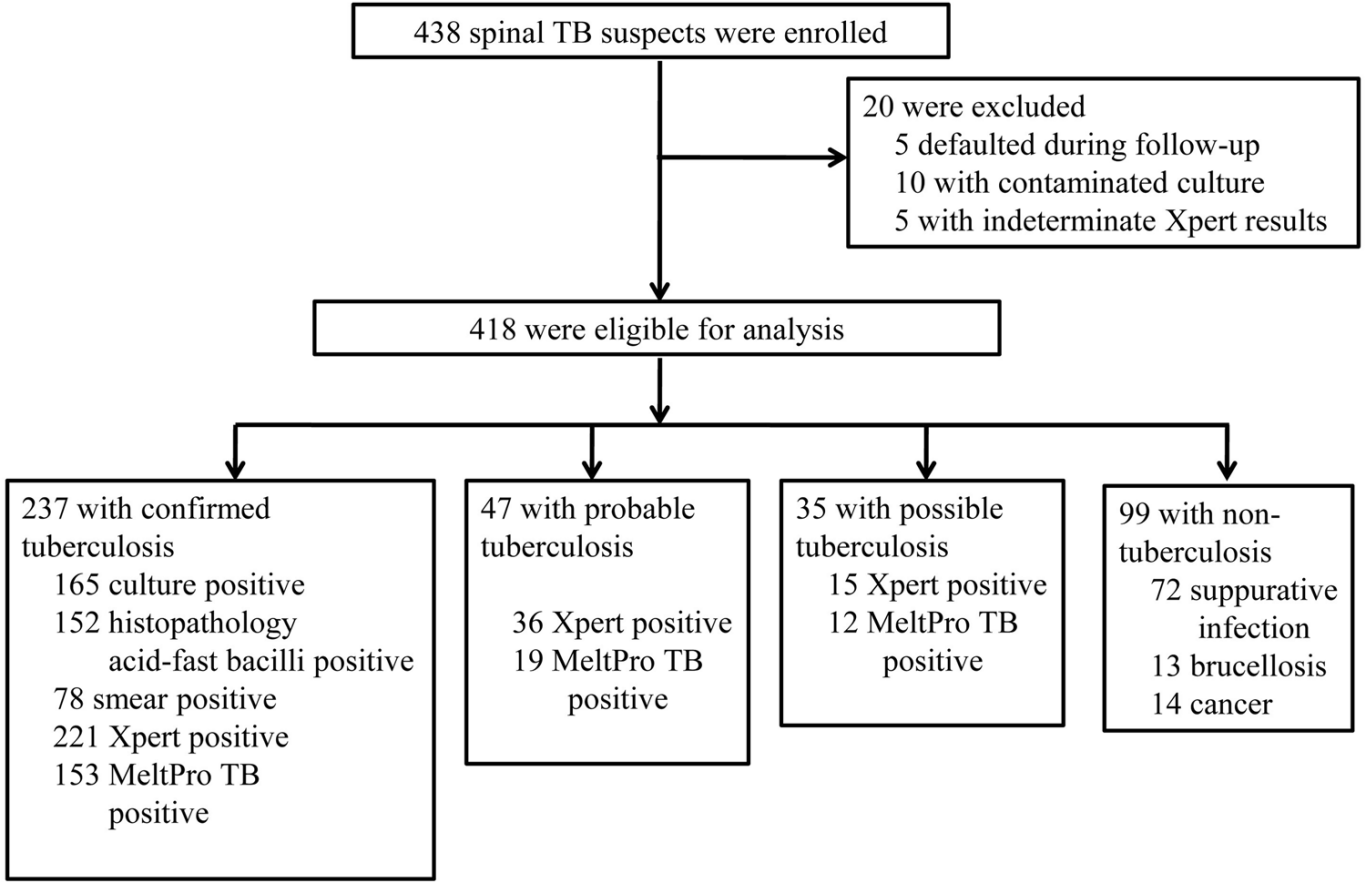
Recruitment and diagnostic classifications of participants

A total of 438 spinal TB suspects were recruited and provided eligible specimens. In total 20 participants were excluded from the analysis, as 5 defaulted during the follow-up term, 10 had a contaminated culture and 5 had indeterminate Xpert results (Fig. 1). Thus, the final sample size for analysis was 418 patients, which included 319 (76.32%) diagnosed spinal TB cases according to the CRS criteria (Fig. 1). The other 99 (23.68%) were classified as “non-TB” patients, including 72 suppurative infection, 13 brucellosis and 14 cancer cases. All of the patients were HIV-uninfected. Basic characteristics stratified by hospital are shown in Table 1.

**Table 1:** Characteristics of study participants stratified by hospitals

**Characteristics**	**Overall**	**Hospital 1**	**Hospital 2**	**Hospital 3**	**Hospital 4**
Total	418	198	134	47	40
Age, median (range), yr	48(13–86)	48(16–86)	48(13–84)	44(20–84)	47(22–81)
Gender					
Male	227(54.31)	116(68.59)	68(50.75)	24(51.06)	19(47.50)
Female	191(45.69)	82(41.41)	66(49.25)	23(48.94)	21(52.50)
Underlying condition					
Diabetes mellitus	51(12.20)	24(12.12)	20(14.93)	4(8.51)	3(7.5)
Hypertension	91(21.77)	45(22.73)	30(22.39)	10(21.28)	6(15.00)
Chronic kidney disease	21(5.02)	5(2.53)	9(6.72)	4(8.51)	3(7.50)
Rheumatologic disease	4(0.96)	2(1.01)	2(1.49)	0	0
Combined TB					
Pulmonary infection	124(29.67)	54(27.27)	46(34.33)	15(31.91)	9(22.50)
Meningitis	6(1.44)	2(1.01)	2(1.49)	1(2.13)	1(2.50)
Pleurisy	25(5.98)	10(5.05)	10(7.46)	4(8.51)	1(2.50)
Infected site of the spinal TB	319(76.32)	148(74.75)	107(79.85)	40(85.11)	24(60.00)
Cervical vertebrae	9(2.82)	3(2.03)	3(2.80)	2(5.00)	1(4.17)
Thoracic vertebrae	152(47.65)	74(50.00)	51(47.66)	16(40.00)	11(45.83)
Lumbar	158(49.53)	71(47.97)	53(49.53)	22(55.00)	12(50.00)

### Performance of different methods compared with culture

Among the 319 spinal TB patients, 78 were positive by the smear test, 165 by any culture method, 184 by MeltPro TB, 233 by pathological examination, and 272 by Xpert assay (Table 2). Notably, the 152 (65.24%, 152/233) cases identified by pathological examinations acquired bacteriological evidence. Among the 165 culture-positive cases, the sensitivities of smear, Xpert, MeltPro TB and histopathology assays were 36.36%, 98.79%, 69.70%, and 84.24%, respectively. Stratified analysis of culture-positive cases according to the smear status was conducted. As expected, the sensitivity in the smear-negative samples was lower than the smear-positive samples. However, there was no significant difference between smear-negative and smear-positive samples (data not shown). Although pathological examination missed 26 culture-positive patients, it detected 94 more cases from the culture-negative but CRS-defined spinal TB cases. The Xpert assay showed even better sensitivity: it missed only two culture-positive specimens but detected 109 more cases. Another molecular test, MeltPro TB, failed to detect 50 culture-positive specimens but produced positive outcomes from 69 culture-negative specimens.

**Table 2:** Diagnostic efficiencies of different methods compared to culture

**Methods**	**All culture positive**	**Culture positive smear positive**	**Culture positive smear negative**
Smear	60/165(36.36)	—	—
Xpert	163/165(98.79)	59/60(98.33)	104/105(99.05)
MeltPro TB	115/165(69.70)	43/60(71.67)	72/105(68.57)
Histopathology	139/165(84.24)	54/60(90.00)	85/105(80.95)
MeltPro TB+histopathology	156/165(94.55)	58/60(96.67)	98/105(93.33)
Xpert+histopathology	164/165(99.39)	60/60(100.00)	104/105(99.05)

### Performance of different methods compared to CRS

**Fig. 2:**
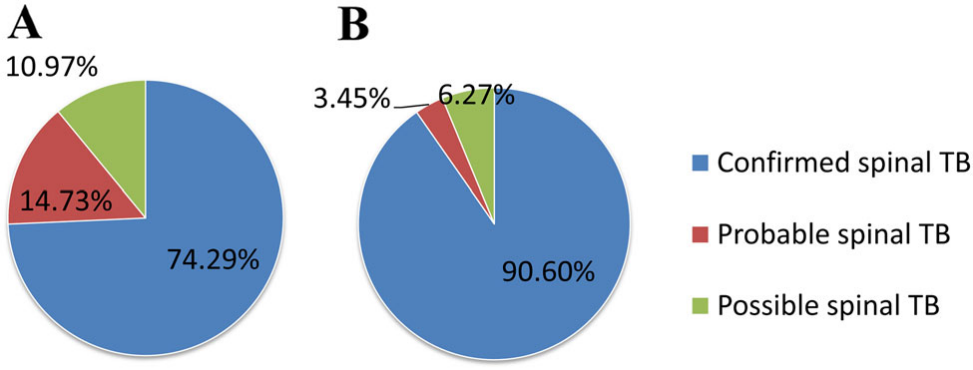
The variation of composite reference standard category composition according to conventional testing only or combined with the Xpert assay. **a** Conventional testing only. **b** Conventional testing combined with the Xpert assay

Two types of CRS criteria were applied: one only referred to the conventional bacteriological testing outcomes, as generally done, whereas the other further included Xpert assay outcomes. Based on the conventional CRS criteria, 237 (74.29%) participants were classified as having confirmed spinal TB (with at least one positive outcome from smear, culture or histopathological AFB examination), 47 (14.73%) probable spinal TB and 35 (10.97%) possible spinal TB. When Xpert assay outcomes were also integrated, the total number of spinal TB patients remained 319, but the percentage of confirmed spinal TB patients showed an obvious increase from 74.29 to 90.6%. Furthermore, 36 out of the 47 (76.60%) probable spinal TB cases were re-classified as confirmed cases, and 16 out of the 35 probable spinal TB cases (45.71% of 35) were re-classified as confirmed cases as well (Fig. 2).

Among the 319 spinal TB patients diagnosed according to CRS, Xpert (85.27%, 272/319) produced a higher sensitivity than histopathology (73.04%, 233/319, *χ*^2^ = 14.448, *P* <0.001), MeltPro TB (57.68%,184/319, *χ*^2^ = 59.532, *P* < 0.001), culture (51.72%,165/319, *χ*^2^ = 83.159, *P* < 0.001) and smear (24.45%,78/319, *χ*^2^ = 238.212, *P* < 0.001) (Table 3). The specificities of histopathological examination and MeltPro TB were 93.94% (93/99) and 98.99% (98/99), respectively. The specificities of smear, culture and Xpert were all 100% (99/99). In total 6 brucellosis cases were misdiagnosed as spinal TB by histopathology, and 1 brucellosis case was misdiagnosed as spinal TB by MeltPro TB. The value of concomitant use of different tests was also considered. Xpert plus culture (85.89%), Xpert plus histopathology (91.22%), and Xpert plus culture plus histopathology (91.54%) demonstrated incremental sensitivity.

**Table 3:** Diagnostic accuracies of all methods compared to the composite reference standard

**Methods**	**Sensitivity**	**Specificity (non-tuberculosis)**	**PPV**	**NPV**
Smear	78/319(24.45)	99/99(100)	78/78(100)	99/340(29.12)
Culture	165/319(51.72)	99/99(100)	165/165(100)	99/353(28.05)
Xpert	272/319(85.27)	99/99(100)	272/272(100)	99/146(67.81)
MeltPro TB	184/319(57.68)	98/99(98.99)	184/185(99.46)	98/233(42.06)
Histopathology	233/319(73.04)	93/99(93.94)	233/239(97.49)	93/179(51.96)
Culture + smear	183/319(57.37)	99/99(100)	183/183(100)	99/235(42.13)
Culture + MeltPro TB	234/319(73.35)	98/99(98.99)	234/235(99.57)	98/183(53.55)
Culture + Xpert	274/319(85.89)	99/99(100)	274/274(100)	99/144(68.75)
Culture + histopathology	259/319(81.19)	93/99(93.94)	259/265(97.74)	93/153(60.78)
MeltPro TB + histopathology	273/319(85.58)	92/99(92.93)	273/280(97.50)	92/138(66.67)
Xpert + histopathology	291/319(91.22)	93/99(93.94)	291/297(97.98)	93/121(76.86)
Culture + MeltPro TB + histopathology	282/319(88.40)	92/99(92.93)	282/289(97.58)	92/129(71.32)
Culture + Xpert + histopathology	292/319(91.54)	93/99(93.94)	292/298(97.99)	93/120(77.50)

**Fig. 3:**
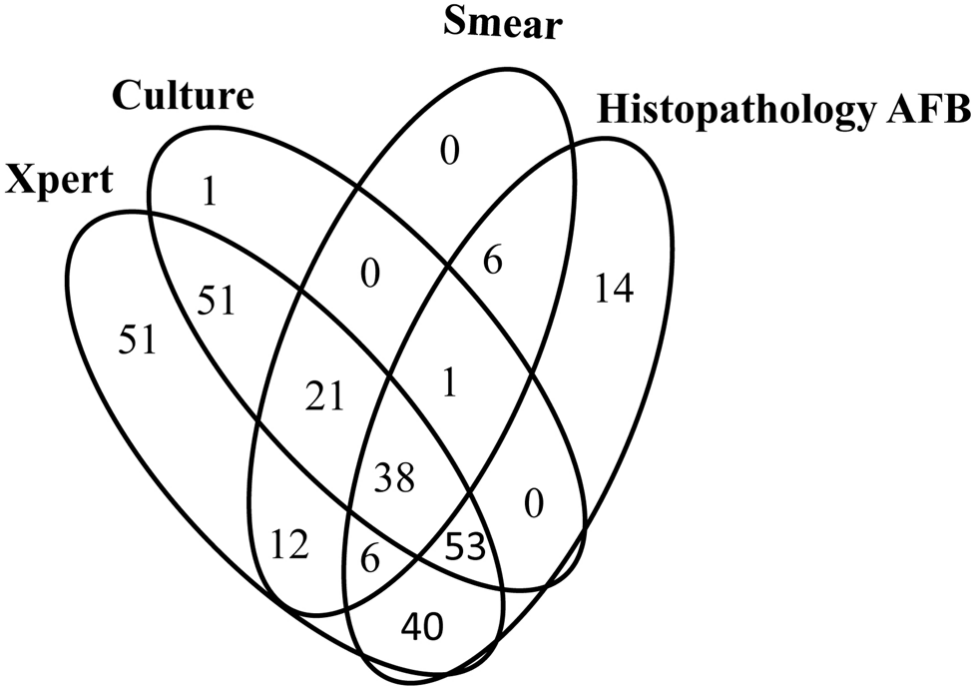
Venn diagram of the overlap of the methods for spinal TB diagnostics

A total of 289 patients had evidence of Mtb, which meant that at least one of this panel of tests produced a positive outcome. Among those 289 bacteriologically confirmed patients, 51 were detected by Xpert assay only, compared to 0, 1, and 14 for smear, culture and pathological examination, respectively (Fig. 3). Surprisingly, one smear-positive, culture-positive, and histopathology positive specimen was missed by the Xpert assay due to unknown reasons.

### Drug resistance detection

Phenotypic DST was performed for the 102 *M. tuberculosis* isolates recovered. Among them, all the corresponding specimens yielded positive Xpert assay outcomes, whereas only 69 were detected by MeltPro TB successfully. Twenty RIF-resistant isolates and 20 INH-resistant isolates were defined by phenotypic DST; 18 of them were multidrug-resistant TB (MDR-TB, defined as resistance to at least RIF and INH) isolates. Xpert was 100% concordant with the phenotypic DST with regard to RIF resistance detection. Among the 69 patients with positive MeltPro TB outcomes, the sensitivity and specificity were, respectively, 100% (20/20) and 97.96% (48/49) for RIF detection and 95.00% (19/20) and 93.88% (46/49) for INH resistance detection.

### Median time to diagnosis

**Fig. 4:**
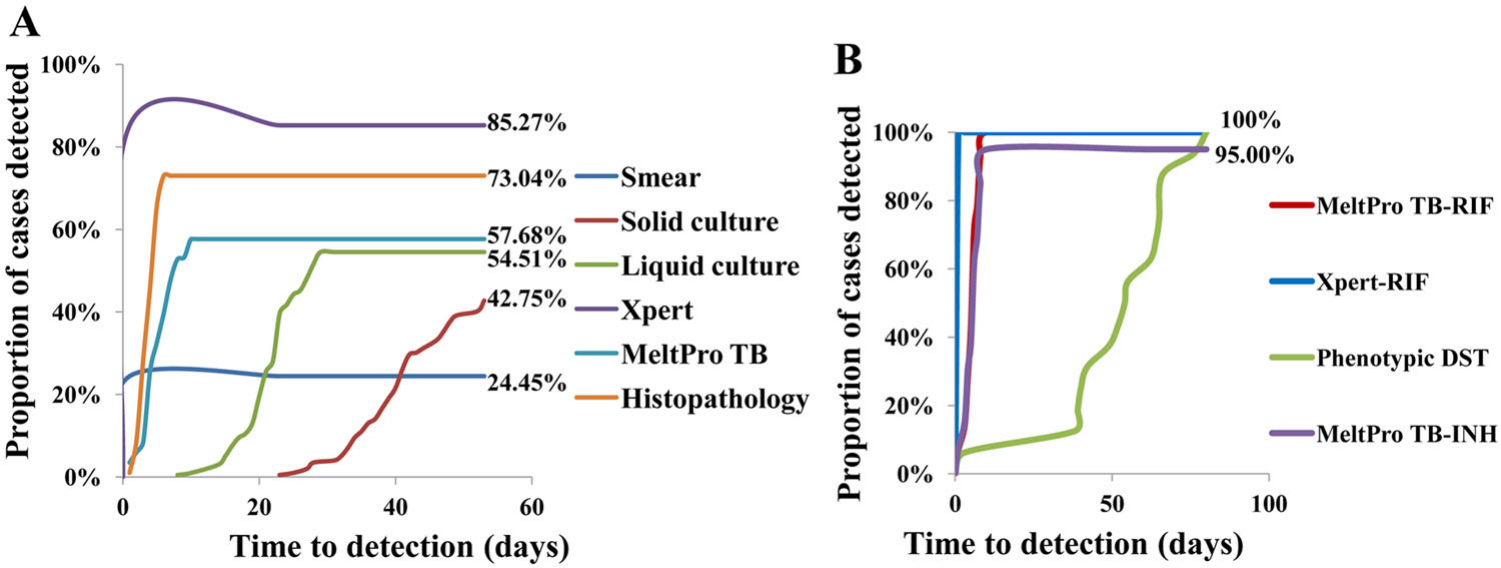
Proportion of tuberculosis cases detected by each method. **a** Tuberculosis case detection. **b** Drug resistance detection. RIF rifampicin, INH isoniazid

The median time to diagnose spinal TB was 1 day (interquartile range, IQR 0–1) for smear microscopy, 50 days (IQR 42–56) for solid culture, 32 days (IQR 23–42) for liquid culture, 1 day (IQR 0–1) for Xpert, 5 days (IQR 4–7) for MeltPro TB and 5 days (IQR 3–6) for histopathological examination (Fig. 4a). The median time to drug resistance detection was 1 day (IQR 0–1) for Xpert, 6 days (IQR 4–7) for MeltPro TB and 53 days (IQR 42–65) for phenotypic DST (Fig. 4b).

## Discussion

Spinal TB has a high chance of neurological sequela if left untreated^5^. Early diagnosis is the most critical step for starting treatment in time. However, in high-burden countries, conventional diagnostic tools are still the mainstay for case findings. More often than not, when conventional testing produces negative results for suspected TB patients, diagnosis and treatment are postponed. Application of newer molecular diagnostic tools has improved the success in finding both pulmonary TB and EPTB greatly. This study is the first prospective, head-to-head comparison of a wide range of diagnostic tests performed on the same specimen collected from spinal TB suspects in a high-burden country. A follow-up period of at least 18 months ensured reliable diagnoses and increased the objectivity of the conclusions.

The efficiency of the Xpert assay has been extensively validated previously, and the technique has been rolled out in many countries^22^. Consistent with previous reports, the Xpert assay demonstrated obvious superiority over conventional tests and MeltPro TB regarding spinal TB diagnosis in this study. In 2016, besides smear and culture, the WHO suggested defining a bacteriologically confirmed case by incorporating results from WHO-approved rapid diagnostics (WRD), such as Xpert^23^. In our assay, the Xpert assay greatly changed the composition percentage of different CRS-defined categories. Based on conventional testing, 74.29% of the CRS-defined cases were classified as confirmed spinal TB. This percentage increased to 90.60% with the integration of Xpert outcomes. Owing to the point of care (POC) feature of the Xpert assay and the advantage of detecting possible MDR patients simultaneously, a timely Xpert assay may change the algorithm of spinal TB patient care. Furthermore, 85.2% (272/319) of the CRS-defined spinal TB patients yielded positive Xpert outcomes, reflecting a high chance of Mtb within pus and the imperfection of culture for bacilli recovery. Because of the high sensitivity of the Xpert assay, concatenating it with other tests did not show any obvious benefit. The sensitivity increased from 85.2 to 91.22% when combining the Xpert assay with pathological examination, but as a trade-off, the specificity dropped from 100 to 93.94% (Table 3).

Among all the bacteriologically confirmed patients, while smear, culture and pathological examination demonstrated complementary efficacy for AFB detection, the Xpert assay did not present this characteristic. The Xpert assay alone identified all the smear-positive, 163/165 culture-positive and 218/233 pathologically diagnosed spinal TB patients. Among the 289 bacteriologically confirmed patients, 51 were detected by the Xpert assay alone, compared to 0, 1, and 14 for smear, culture and pathological examination, respectively. Xpert-positive results without other bacteriological supporting evidence can raise concerns about false-positive outcomes. Our previous work found that 98.77% (80/81) of specimens collected from bone and joint TB were confirmed as TB or were strongly suggestive of TB by pathological examination^24^. Therefore, these Xpert-positive-only results were true positives. Our outcomes highlight the value of the Xpert assay as a frontline technique for spinal TB suspects in clinical practice. However, the price of the cartridge and the equipment for the Xpert assay will definitely prevent patients who cannot afford the expense from benefitting from the assay.

A previous study showed that MeltPro TB had very good accuracy for PTB diagnosis using sputum^19^. In our study, Xpert performed much better than MeltPro TB. Particularly, the sensitivity was substantially greater (85.27% vs 57.68% compared with CRS, 98.79% vs 69.70% compared with culture). The obviously inferior sensitivity of MeltPro TB might reflect the low efficiency of DNA abstraction from pus specimens. The MeltPro TB assay requires some manual steps for DNA preparation, and the different characteristics of pus from sputum may hurt the efficiency. Although less sensitive than Xpert, MeltPro TB was more sensitive and rapid than culture (63.67% vs. 57.09% among bacteriologically confirmed spinal TB). Furthermore, besides RIF and INH, it can also detect resistance to other drugs, such as streptomycin, ethambutol and fluoroquinolones, within a few hours, which greatly enhances its value. Although the median turn-around time for MeltPro TB was 5–6 days in this assay, this mainly reflected the routine work arrangement, while the actual time needed for the assay was <3 h.

Spinal TB is a localized manifestation of a systemic disease. Anti-tuberculosis chemotherapy plays a fundamental role in spinal TB treatment. The emergence of drug-resistant TB, especially MDR-TB, is considered the greatest obstacle to global TB control due to the difficulties of diagnosis and treatment^25^. As our and other studies have demonstrated, conventional phenotypic DST methods take 2–3 months from sample collection to outcome, while Xpert and MeltPro TB could find drug-resistant patients within a few hours by identifying mutations conferring resistance, which dramatically diminishes the delay of appropriate treatment. In this study, the Xpert assay had 100% consistency with the phenotypic DST in RIF resistance detection. MeltPro TB detected all of the RIF-resistant cases as well, but it classified one out of 49 RIF-sensitive strains as resistant. Regarding INH resistance detection, the sensitivity and specificity of MeltPro TB were 95.00% and 93.88%, respectively. Considering their accuracy, convenience, and rapid turnaround time, Xpert and MeltPro TB could improve the diagnosis and treatment of drug-resistant spinal TB. Application of these rapid molecular tests can shorten the whole course of medical care and decrease the risk of disabling sequelae caused by spinal TB. An updated laboratory testing algorithm of spinal TB is desirable.

The prospective multi-center design, the ≥18 months of follow-up and the large sample size were important strengths of this study, but its limitations should be noted. First, this study was performed in a high-burden country, so the performances of different tests for spinal TB diagnosis in different settings might be variable. Second, the incidence of brucellosis could have affected the specificity of some tests. This study was performed in northern China, where brucellosis was endemic^26^, which evidently impacted the specificity of the pathological examinations.

In conclusion, new molecular tests demonstrated excellent efficiency for spinal TB diagnosis in this cohort study. Their implementation as the initial diagnostic tests could greatly increase the proportion of confirmed cases and dramatically reduce the delay of appropriate treatment. Spinal TB does not entail a paucity of bacilli, as other extrapulmonary TB types do, and new molecular tests reinforce this fact.

## Materials and methods

### Study design and participants

Pus specimens were prospectively collected in a cohort study (“The Optimal Duration of Preoperative Anti-tuberculosis Treatment of Spinal Tuberculosis” (NCT02477852)) to identify the appropriate algorithm for spinal TB patient care, and all the patients were followed up for a minimum of 18 months. Adults with suspected spinal TB were enrolled consecutively in four hospitals in China (Beijing Chest Hospital, 309th Hospital of Chinese PLA, Beijing Geriatric Hospital, and PLA Army General Hospital) from June 2014 to June 2016. Patients were enrolled in this diagnostic evaluation study after a detailed clinical history that included radiological and histopathological examination reports, no previous treatments with any anti-TB drugs and no receipt of anti-TB treatment within the previous 2 weeks, along with a minimum specimen volume of 4 ml. Each specimen was subjected to all the microbiological tests and pathological examinations. The study was approved by the Ethics Committee of the Beijing Chest Hospital, Capital Medical University, and written informed consent was obtained from each participant.

### Patient categories

The composite reference standard (CRS) of this study was composed of clinical findings, laboratory smear and culture outcomes (both solid culture and liquid culture), histopathological reports, radiological imaging, and at least 18 months of follow-up since the date of enrollment. Another criterion of CRS included the application and interpretation of Xpert assay outcomes. Based on the CRS, patients were categorized into 4 groups: (1) Confirmed spinal TB: bacteriological evidence was acquired; (2) Probable spinal TB: bacteriological evidence was negative; clinical symptoms, radiological findings, and histopathological examinations were suggestive of TB; (3) Possible spinal TB: bacteriological evidence was negative; clinical symptoms were suggestive of TB; histopathology examination and/or radiological examination were mildly suggestive of TB; the patient responded well to the empirical anti-tubercular treatment during the follow-up term; and (4) Non-TB: diagnosed as other diseases, or the laboratory testing was not suggestive of TB, and the patient improved without receiving anti-tubercular treatment.

### Conventional bacteriological testing

Direct smear was prepared and stained with auramine and examined by light-emitting diode microscopy. The smear was read and interpreted in accordance with WHO guidelines^27^. After processing with N-acetyl-L-cysteine and sodium hydroxide (NALC-NaOH) and centrifugation, the resuspended pellet was subjected to cultivation on both solid Lowenstein–Jensen medium (Encode Medical Engineering Co., Ltd, China) and a liquid medium mycobacteria growth indicator tube (MGIT) 960 system (Becton, Dickinson and Company, USA). For all the isolates, MPT64 antigen testing was performed to confirm the presence of *M. tuberculosis* complex.

### Pathological examination

The tissue specimens were fixed in neutral formalin, dehydrated and subsequently paraffin-embedded. Four-micrometer sections were stained with hematoxylin and eosin solution and observed by light microscopy for patho-morphological changes. Acid-fast bacilli (AFB) or its DNA were detected from the fixed specimens either by ZN staining or by molecular testing as described before^24^. The pathological diagnostic categories included (1) Confirmed TB: chronic granulomatous inflammation with or without caseous necrosis were observed, and AFB or its DNA were detected in the lesion; (2) Suggestive of TB: typical chronic granulomatous inflammation with caseous necrosis was observed, but bacteriological examination was negative; and (3) Non-TB: neither granulomatous inflammation nor caseous necrosis was observed, and no bacteriological evidence was present.

### Molecular testing

The Xpert assay was performed as per the manufacturer’s instructions. Briefly, 1 ml of specimen was mixed with 2 ml of Xpert sample-processing reagent, vortexed for at least 10 s, and then incubated at room temperature for 10 min. The mixture was again vortexed for 10 s and incubated at room temperature for 5 min. A total of 2 ml of the mixture was transferred into an Xpert cartridge and loaded into a GeneXpert instrument. The automatic detection procedure was then run. The MeltPro TB assay was conducted following the manufacturer’s instructions. Briefly, crude DNA was extracted from a 1 ml aliquot of the decontaminated specimens with an automatic DNA extraction machine (Zeesan Biotecheh, Xiamen, China) using the paramagnetic particle method. The program for amplification and the melting curve were analyzed on a LightCycler 480 system (Roche Applied Science, Indianapolis, USA). The fluorescent signal intensity was collected at FAM and TET channels. The Tm values were obtained by identifying the peaks of the melting curves.

### Drug susceptibility testing

Drug susceptibility testing (DST) was performed with the proportion method using Lowenstein–Jensen medium. The critical concentration used was 0.2 μg/ml for isoniazid (INH) and 40 μg/ml for rifampicin (RIF).

### Statistical analyses

The statistical analysis was conducted using SPSS (version 19.0 software, IBM, Armonk, NY, USA). The sensitivity, specificity, positive predictive value (PPV) and negative predictive value (NPV) of different assays were calculated. Differences in the categorical variables were evaluated using a two-tailed Fisher exact test. Differences were considered statistically significant at *P* < 0.05.
